# Supplementary material for: Modeling glioblastoma heterogeneity as a dynamic network of cell states
Source: Mol Syst Biol. 2021 Sep 16;17(9):e10105. doi: 10.15252/msb.202010105 (PMC8444284; doi:10.15252/msb.202010105)
Supplement: Supplementary file 6 — Source Data for Figure 5 [file MSB-17-e10105-s004.zip › Figure5A_sourcedata/GSEA_3071/nosigdb_stateII.GseaPreranked.1622795151171/RICHARDS_NOWAKOWSKI_SCIENCE_2017_IPC_DIV1_UPREG.html]

Details for gene set RICHARDS\_NOWAKOWSKI\_SCIENCE\_2017\_IPC\_DIV1\_UPREG[GSEA]

|  || Dataset | state23071 |
| Phenotype | NoPhenotypeAvailable |
| Upregulated in class | na\_pos |
| GeneSet | RICHARDS\_NOWAKOWSKI\_SCIENCE\_2017\_IPC\_DIV1\_UPREG |
| Enrichment Score (ES) | 0.4750549 |
| Normalized Enrichment Score (NES) | 3.6488864 |
| Nominal p-value | 0.0 |
| FDR q-value | 0.0 |
| FWER p-Value | 0.0 |
Table: GSEA Results Summary

  

Fig 1: Enrichment plot: RICHARDS\_NOWAKOWSKI\_SCIENCE\_2017\_IPC\_DIV1\_UPREG      
 Profile of the Running ES Score & Positions of GeneSet Members on the Rank Ordered List

  

| PROBE | GENE SYMBOL | GENE\_TITLE | RANK IN GENE LIST | RANK METRIC SCORE | RUNNING ES | CORE ENRICHMENT || 1 | HIST1H1D |  |  | 0 | 2.323 | 0.0072 | Yes |
| 2 | CLSPN |  |  | 1 | 1.984 | 0.0134 | Yes |
| 3 | HIST1H4C |  |  | 2 | 1.902 | 0.0193 | Yes |
| 4 | RRM2 |  |  | 3 | 1.857 | 0.0250 | Yes |
| 5 | ANLN |  |  | 4 | 1.760 | 0.0305 | Yes |
| 6 | HIST1H1B |  |  | 7 | 1.669 | 0.0351 | Yes |
| 7 | TYMS |  |  | 8 | 1.665 | 0.0403 | Yes |
| 8 | MCM10 |  |  | 9 | 1.654 | 0.0454 | Yes |
| 9 | CDK1 |  |  | 14 | 1.540 | 0.0491 | Yes |
| 10 | DIAPH3 |  |  | 15 | 1.531 | 0.0539 | Yes |
| 11 | ZWINT |  |  | 16 | 1.510 | 0.0586 | Yes |
| 12 | HJURP |  |  | 17 | 1.490 | 0.0632 | Yes |
| 13 | ATAD5 |  |  | 18 | 1.469 | 0.0677 | Yes |
| 14 | BRCA2 |  |  | 22 | 1.445 | 0.0714 | Yes |
| 15 | UBE2C |  |  | 23 | 1.438 | 0.0759 | Yes |
| 16 | CENPU |  |  | 24 | 1.416 | 0.0803 | Yes |
| 17 | RAD51AP1 |  |  | 25 | 1.384 | 0.0846 | Yes |
| 18 | CENPK |  |  | 26 | 1.382 | 0.0888 | Yes |
| 19 | FBXO5 |  |  | 30 | 1.359 | 0.0922 | Yes |
| 20 | USP1 |  |  | 31 | 1.357 | 0.0965 | Yes |
| 21 | DTL |  |  | 33 | 1.330 | 0.1003 | Yes |
| 22 | RRM1 |  |  | 37 | 1.310 | 0.1036 | Yes |
| 23 | ORC6 |  |  | 39 | 1.308 | 0.1073 | Yes |
| 24 | CDCA5 |  |  | 41 | 1.289 | 0.1111 | Yes |
| 25 | TK1 |  |  | 42 | 1.281 | 0.1150 | Yes |
| 26 | AURKB |  |  | 44 | 1.276 | 0.1187 | Yes |
| 27 | HELLS |  |  | 46 | 1.259 | 0.1224 | Yes |
| 28 | MCM8 |  |  | 48 | 1.253 | 0.1260 | Yes |
| 29 | BRCA1 |  |  | 49 | 1.249 | 0.1299 | Yes |
| 30 | CCNA2 |  |  | 53 | 1.230 | 0.1329 | Yes |
| 31 | NEIL3 |  |  | 54 | 1.228 | 0.1367 | Yes |
| 32 | TICRR |  |  | 55 | 1.227 | 0.1405 | Yes |
| 33 | DNAJC9 |  |  | 56 | 1.217 | 0.1443 | Yes |
| 34 | SKA3 |  |  | 57 | 1.215 | 0.1480 | Yes |
| 35 | PCNA |  |  | 58 | 1.214 | 0.1518 | Yes |
| 36 | HIST1H1C |  |  | 60 | 1.206 | 0.1553 | Yes |
| 37 | MAD2L1 |  |  | 61 | 1.199 | 0.1590 | Yes |
| 38 | UBE2T |  |  | 62 | 1.192 | 0.1627 | Yes |
| 39 | ATAD2 |  |  | 63 | 1.189 | 0.1664 | Yes |
| 40 | RFC3 |  |  | 67 | 1.157 | 0.1691 | Yes |
| 41 | SPC25 |  |  | 68 | 1.156 | 0.1727 | Yes |
| 42 | MELK |  |  | 69 | 1.145 | 0.1763 | Yes |
| 43 | TOP2A |  |  | 72 | 1.132 | 0.1793 | Yes |
| 44 | KIF2C |  |  | 73 | 1.132 | 0.1828 | Yes |
| 45 | UHRF1 |  |  | 79 | 1.113 | 0.1848 | Yes |
| 46 | FAM111A |  |  | 80 | 1.112 | 0.1883 | Yes |
| 47 | GMNN |  |  | 83 | 1.108 | 0.1912 | Yes |
| 48 | CEP55 |  |  | 87 | 1.096 | 0.1938 | Yes |
| 49 | KIFC1 |  |  | 88 | 1.094 | 0.1972 | Yes |
| 50 | SKA1 |  |  | 91 | 1.078 | 0.2000 | Yes |
| 51 | XRCC2 |  |  | 92 | 1.076 | 0.2033 | Yes |
| 52 | VRK1 |  |  | 93 | 1.074 | 0.2066 | Yes |
| 53 | NDC80 |  |  | 94 | 1.074 | 0.2100 | Yes |
| 54 | MASTL |  |  | 96 | 1.063 | 0.2130 | Yes |
| 55 | TMPO |  |  | 99 | 1.056 | 0.2157 | Yes |
| 56 | NCAPG |  |  | 100 | 1.056 | 0.2190 | Yes |
| 57 | MYBL1 |  |  | 102 | 1.043 | 0.2220 | Yes |
| 58 | NF2 |  |  | 103 | 1.036 | 0.2252 | Yes |
| 59 | MND1 |  |  | 104 | 1.036 | 0.2284 | Yes |
| 60 | MKI67 |  |  | 107 | 1.031 | 0.2311 | Yes |
| 61 | STIL |  |  | 108 | 1.031 | 0.2343 | Yes |
| 62 | BRIP1 |  |  | 110 | 1.029 | 0.2372 | Yes |
| 63 | TPX2 |  |  | 111 | 1.025 | 0.2404 | Yes |
| 64 | CDCA8 |  |  | 113 | 1.018 | 0.2432 | Yes |
| 65 | KIF11 |  |  | 115 | 1.014 | 0.2461 | Yes |
| 66 | NCAPH |  |  | 116 | 1.013 | 0.2493 | Yes |
| 67 | NCAPD3 |  |  | 117 | 1.013 | 0.2524 | Yes |
| 68 | GJC1 |  |  | 121 | 1.007 | 0.2547 | Yes |
| 69 | SMC2 |  |  | 123 | 1.005 | 0.2575 | Yes |
| 70 | NUF2 |  |  | 125 | 1.004 | 0.2604 | Yes |
| 71 | DEPDC1 |  |  | 126 | 0.999 | 0.2635 | Yes |
| 72 | CHAF1A |  |  | 127 | 0.999 | 0.2666 | Yes |
| 73 | CKS1B |  |  | 128 | 0.999 | 0.2697 | Yes |
| 74 | TACC3 |  |  | 130 | 0.992 | 0.2725 | Yes |
| 75 | CENPL |  |  | 132 | 0.989 | 0.2753 | Yes |
| 76 | KIF20B |  |  | 133 | 0.989 | 0.2784 | Yes |
| 77 | FANCI |  |  | 138 | 0.984 | 0.2803 | Yes |
| 78 | WDR76 |  |  | 142 | 0.975 | 0.2825 | Yes |
| 79 | ESCO2 |  |  | 144 | 0.971 | 0.2853 | Yes |
| 80 | CKAP2L |  |  | 148 | 0.959 | 0.2874 | Yes |
| 81 | FANCB |  |  | 151 | 0.956 | 0.2898 | Yes |
| 82 | BLM |  |  | 156 | 0.942 | 0.2917 | Yes |
| 83 | NCAPG2 |  |  | 159 | 0.937 | 0.2940 | Yes |
| 84 | KIF23 |  |  | 161 | 0.936 | 0.2966 | Yes |
| 85 | SHCBP1 |  |  | 164 | 0.933 | 0.2990 | Yes |
| 86 | RTKN2 |  |  | 166 | 0.931 | 0.3016 | Yes |
| 87 | GTSE1 |  |  | 167 | 0.929 | 0.3045 | Yes |
| 88 | CEP78 |  |  | 170 | 0.927 | 0.3068 | Yes |
| 89 | CDCA2 |  |  | 171 | 0.922 | 0.3097 | Yes |
| 90 | CENPM |  |  | 178 | 0.901 | 0.3108 | Yes |
| 91 | E2F7 |  |  | 179 | 0.901 | 0.3136 | Yes |
| 92 | PIM1 |  |  | 184 | 0.896 | 0.3153 | Yes |
| 93 | ZNF850 |  |  | 187 | 0.888 | 0.3175 | Yes |
| 94 | FAM83D |  |  | 197 | 0.876 | 0.3178 | Yes |
| 95 | SPDL1 |  |  | 199 | 0.874 | 0.3202 | Yes |
| 96 | C21orf58 |  |  | 201 | 0.873 | 0.3226 | Yes |
| 97 | PLK4 |  |  | 205 | 0.870 | 0.3245 | Yes |
| 98 | PRIM2 |  |  | 212 | 0.865 | 0.3255 | Yes |
| 99 | H2AFZ |  |  | 213 | 0.864 | 0.3282 | Yes |
| 100 | PBK |  |  | 214 | 0.864 | 0.3309 | Yes |
| 101 | CEP152 |  |  | 217 | 0.860 | 0.3330 | Yes |
| 102 | CDCA3 |  |  | 219 | 0.858 | 0.3354 | Yes |
| 103 | RFC5 |  |  | 222 | 0.851 | 0.3375 | Yes |
| 104 | BUB1B |  |  | 223 | 0.851 | 0.3402 | Yes |
| 105 | ASPM |  |  | 231 | 0.842 | 0.3408 | Yes |
| 106 | EZH2 |  |  | 233 | 0.839 | 0.3432 | Yes |
| 107 | MIS18A |  |  | 239 | 0.833 | 0.3444 | Yes |
| 108 | DBF4 |  |  | 240 | 0.832 | 0.3470 | Yes |
| 109 | RACGAP1 |  |  | 241 | 0.831 | 0.3496 | Yes |
| 110 | PRC1 |  |  | 242 | 0.830 | 0.3521 | Yes |
| 111 | TUBB6 |  |  | 244 | 0.828 | 0.3544 | Yes |
| 112 | KNTC1 |  |  | 249 | 0.825 | 0.3559 | Yes |
| 113 | LMNB1 |  |  | 251 | 0.824 | 0.3582 | Yes |
| 114 | CHEK1 |  |  | 252 | 0.822 | 0.3607 | Yes |
| 115 | KIF18B |  |  | 254 | 0.815 | 0.3630 | Yes |
| 116 | DNA2 |  |  | 263 | 0.797 | 0.3633 | Yes |
| 117 | DKC1 |  |  | 268 | 0.789 | 0.3646 | Yes |
| 118 | CENPI |  |  | 269 | 0.788 | 0.3671 | Yes |
| 119 | TUBB4B |  |  | 271 | 0.784 | 0.3692 | Yes |
| 120 | HMGB2 |  |  | 276 | 0.780 | 0.3705 | Yes |
| 121 | TUBA1C |  |  | 277 | 0.779 | 0.3729 | Yes |
| 122 | SMC4 |  |  | 279 | 0.777 | 0.3751 | Yes |
| 123 | CENPE |  |  | 284 | 0.775 | 0.3764 | Yes |
| 124 | RFWD3 |  |  | 290 | 0.769 | 0.3774 | Yes |
| 125 | SMTN |  |  | 292 | 0.766 | 0.3795 | Yes |
| 126 | BARD1 |  |  | 295 | 0.760 | 0.3813 | Yes |
| 127 | DEK |  |  | 302 | 0.754 | 0.3820 | Yes |
| 128 | CENPA |  |  | 303 | 0.751 | 0.3843 | Yes |
| 129 | MMS22L |  |  | 306 | 0.747 | 0.3861 | Yes |
| 130 | CENPO |  |  | 307 | 0.747 | 0.3884 | Yes |
| 131 | KIF4A |  |  | 309 | 0.746 | 0.3905 | Yes |
| 132 | CKS2 |  |  | 312 | 0.745 | 0.3922 | Yes |
| 133 | PRR11 |  |  | 314 | 0.743 | 0.3943 | Yes |
| 134 | CENPN |  |  | 320 | 0.737 | 0.3952 | Yes |
| 135 | CCNF |  |  | 321 | 0.737 | 0.3975 | Yes |
| 136 | CEP128 |  |  | 324 | 0.734 | 0.3992 | Yes |
| 137 | ANP32B |  |  | 325 | 0.734 | 0.4015 | Yes |
| 138 | CENPW |  |  | 330 | 0.727 | 0.4026 | Yes |
| 139 | EBP |  |  | 336 | 0.721 | 0.4035 | Yes |
| 140 | SPC24 |  |  | 340 | 0.719 | 0.4049 | Yes |
| 141 | ARHGAP11A |  |  | 346 | 0.712 | 0.4057 | Yes |
| 142 | LMNB2 |  |  | 352 | 0.709 | 0.4066 | Yes |
| 143 | AURKA |  |  | 360 | 0.705 | 0.4068 | Yes |
| 144 | DTYMK |  |  | 364 | 0.701 | 0.4082 | Yes |
| 145 | TTK |  |  | 365 | 0.701 | 0.4104 | Yes |
| 146 | CENPJ |  |  | 376 | 0.695 | 0.4098 | Yes |
| 147 | KIF14 |  |  | 378 | 0.693 | 0.4116 | Yes |
| 148 | PARPBP |  |  | 382 | 0.688 | 0.4130 | Yes |
| 149 | RFC4 |  |  | 385 | 0.686 | 0.4145 | Yes |
| 150 | KIF18A |  |  | 392 | 0.683 | 0.4150 | Yes |
| 151 | EXOSC9 |  |  | 399 | 0.679 | 0.4155 | Yes |
| 152 | KIF15 |  |  | 404 | 0.676 | 0.4165 | Yes |
| 153 | NUDC |  |  | 407 | 0.673 | 0.4180 | Yes |
| 154 | CCDC34 |  |  | 411 | 0.672 | 0.4193 | Yes |
| 155 | PA2G4 |  |  | 412 | 0.672 | 0.4214 | Yes |
| 156 | TTF2 |  |  | 413 | 0.671 | 0.4234 | Yes |
| 157 | SAE1 |  |  | 418 | 0.669 | 0.4244 | Yes |
| 158 | KPNA2 |  |  | 420 | 0.669 | 0.4262 | Yes |
| 159 | FOXM1 |  |  | 423 | 0.667 | 0.4277 | Yes |
| 160 | RAN |  |  | 424 | 0.667 | 0.4298 | Yes |
| 161 | SMC3 |  |  | 425 | 0.667 | 0.4319 | Yes |
| 162 | PHGDH |  |  | 429 | 0.664 | 0.4331 | Yes |
| 163 | INCENP |  |  | 432 | 0.660 | 0.4346 | Yes |
| 164 | BUB3 |  |  | 441 | 0.657 | 0.4345 | Yes |
| 165 | PKP4 |  |  | 444 | 0.654 | 0.4359 | Yes |
| 166 | PIF1 |  |  | 445 | 0.653 | 0.4380 | Yes |
| 167 | PARP2 |  |  | 451 | 0.650 | 0.4386 | Yes |
| 168 | NCAPD2 |  |  | 457 | 0.647 | 0.4392 | Yes |
| 169 | RAD18 |  |  | 465 | 0.642 | 0.4393 | Yes |
| 170 | EZR |  |  | 466 | 0.642 | 0.4413 | Yes |
| 171 | SNRPB |  |  | 467 | 0.642 | 0.4433 | Yes |
| 172 | DBF4B |  |  | 473 | 0.640 | 0.4439 | Yes |
| 173 | HMMR |  |  | 475 | 0.639 | 0.4456 | Yes |
| 174 | DCTPP1 |  |  | 477 | 0.638 | 0.4473 | Yes |
| 175 | POLH |  |  | 487 | 0.635 | 0.4468 | Yes |
| 176 | FSTL1 |  |  | 495 | 0.630 | 0.4469 | Yes |
| 177 | LMO7 |  |  | 499 | 0.629 | 0.4480 | Yes |
| 178 | RHEB |  |  | 500 | 0.629 | 0.4499 | Yes |
| 179 | BIRC5 |  |  | 505 | 0.628 | 0.4508 | Yes |
| 180 | ECT2 |  |  | 510 | 0.626 | 0.4516 | Yes |
| 181 | IQGAP3 |  |  | 516 | 0.621 | 0.4522 | Yes |
| 182 | GEN1 |  |  | 517 | 0.620 | 0.4541 | Yes |
| 183 | NDC1 |  |  | 521 | 0.617 | 0.4552 | Yes |
| 184 | HAUS8 |  |  | 527 | 0.613 | 0.4557 | Yes |
| 185 | ZGRF1 |  |  | 531 | 0.611 | 0.4568 | Yes |
| 186 | TACC1 |  |  | 534 | 0.610 | 0.4581 | Yes |
| 187 | BTG3 |  |  | 540 | 0.607 | 0.4587 | Yes |
| 188 | DDX39A |  |  | 542 | 0.605 | 0.4603 | Yes |
| 189 | RCC1 |  |  | 549 | 0.603 | 0.4605 | Yes |
| 190 | C18orf54 |  |  | 550 | 0.603 | 0.4624 | Yes |
| 191 | CENPQ |  |  | 569 | 0.590 | 0.4592 | Yes |
| 192 | MIS18BP1 |  |  | 574 | 0.588 | 0.4600 | Yes |
| 193 | CENPF |  |  | 575 | 0.588 | 0.4618 | Yes |
| 194 | CCNB1 |  |  | 579 | 0.587 | 0.4628 | Yes |
| 195 | FAM110A |  |  | 581 | 0.586 | 0.4643 | Yes |
| 196 | TIMELESS |  |  | 591 | 0.583 | 0.4637 | Yes |
| 197 | CEP135 |  |  | 598 | 0.578 | 0.4638 | Yes |
| 198 | EXOSC8 |  |  | 604 | 0.576 | 0.4642 | Yes |
| 199 | SMS |  |  | 608 | 0.575 | 0.4652 | Yes |
| 200 | TUBG1 |  |  | 617 | 0.570 | 0.4648 | Yes |
| 201 | CKAP2 |  |  | 619 | 0.569 | 0.4663 | Yes |
| 202 | CDC25C |  |  | 626 | 0.568 | 0.4664 | Yes |
| 203 | ADCY3 |  |  | 630 | 0.566 | 0.4673 | Yes |
| 204 | SASS6 |  |  | 637 | 0.562 | 0.4674 | Yes |
| 205 | SPAG5 |  |  | 642 | 0.555 | 0.4680 | Yes |
| 206 | NUSAP1 |  |  | 654 | 0.550 | 0.4667 | Yes |
| 207 | FANCM |  |  | 659 | 0.549 | 0.4673 | Yes |
| 208 | CCT5 |  |  | 662 | 0.548 | 0.4685 | Yes |
| 209 | H2AFX |  |  | 671 | 0.544 | 0.4680 | Yes |
| 210 | NUP107 |  |  | 675 | 0.539 | 0.4688 | Yes |
| 211 | TUBB |  |  | 678 | 0.538 | 0.4699 | Yes |
| 212 | SLC25A5 |  |  | 680 | 0.537 | 0.4713 | Yes |
| 213 | CEP85 |  |  | 683 | 0.537 | 0.4724 | Yes |
| 214 | WEE1 |  |  | 685 | 0.536 | 0.4738 | Yes |
| 215 | CIT |  |  | 690 | 0.535 | 0.4744 | Yes |
| 216 | DLGAP5 |  |  | 712 | 0.529 | 0.4703 | Yes |
| 217 | CORO1C |  |  | 718 | 0.526 | 0.4705 | Yes |
| 218 | GPSM2 |  |  | 719 | 0.526 | 0.4722 | Yes |
| 219 | HAUS6 |  |  | 724 | 0.525 | 0.4727 | Yes |
| 220 | C4orf46 |  |  | 733 | 0.521 | 0.4721 | Yes |
| 221 | CCDC18 |  |  | 735 | 0.520 | 0.4734 | Yes |
| 222 | RANGAP1 |  |  | 736 | 0.520 | 0.4751 | Yes |
| 223 | PHF19 |  |  | 745 | 0.518 | 0.4745 | No |
| 224 | LINC01224 |  |  | 760 | 0.513 | 0.4722 | No |
| 225 | PTGES3 |  |  | 763 | 0.512 | 0.4732 | No |
| 226 | POLQ |  |  | 771 | 0.508 | 0.4729 | No |
| 227 | CMC2 |  |  | 785 | 0.503 | 0.4709 | No |
| 228 | DCLRE1C |  |  | 786 | 0.503 | 0.4725 | No |
| 229 | MYC |  |  | 790 | 0.502 | 0.4732 | No |
| 230 | BASP1 |  |  | 802 | 0.497 | 0.4717 | No |
| 231 | CCDC150 |  |  | 804 | 0.496 | 0.4730 | No |
| 232 | SUV39H2 |  |  | 821 | 0.491 | 0.4701 | No |
| 233 | CLMP |  |  | 822 | 0.490 | 0.4716 | No |
| 234 | SRSF10 |  |  | 831 | 0.489 | 0.4709 | No |
| 235 | CDC20 |  |  | 844 | 0.486 | 0.4692 | No |
| 236 | TUBA1B |  |  | 862 | 0.481 | 0.4660 | No |
| 237 | NUP50 |  |  | 888 | 0.473 | 0.4606 | No |
| 238 | CISD2 |  |  | 891 | 0.473 | 0.4615 | No |
| 239 | SUZ12 |  |  | 894 | 0.472 | 0.4624 | No |
| 240 | CEP57L1 |  |  | 901 | 0.471 | 0.4622 | No |
| 241 | DEPDC1B |  |  | 906 | 0.470 | 0.4626 | No |
| 242 | ITGB3BP |  |  | 935 | 0.462 | 0.4563 | No |
| 243 | HIRIP3 |  |  | 936 | 0.462 | 0.4578 | No |
| 244 | TAGLN2 |  |  | 940 | 0.461 | 0.4584 | No |
| 245 | LCLAT1 |  |  | 947 | 0.460 | 0.4581 | No |
| 246 | LIN9 |  |  | 949 | 0.459 | 0.4593 | No |
| 247 | KIF22 |  |  | 975 | 0.450 | 0.4538 | No |
| 248 | FANCD2 |  |  | 976 | 0.450 | 0.4552 | No |
| 249 | LRRC8A |  |  | 994 | 0.446 | 0.4519 | No |
| 250 | TRIM28 |  |  | 1004 | 0.444 | 0.4508 | No |
| 251 | PLK1 |  |  | 1009 | 0.442 | 0.4511 | No |
| 252 | SEC11A |  |  | 1019 | 0.438 | 0.4500 | No |
| 253 | ADRM1 |  |  | 1034 | 0.432 | 0.4475 | No |
| 254 | TBC1D31 |  |  | 1050 | 0.428 | 0.4447 | No |
| 255 | DLEU2 |  |  | 1059 | 0.426 | 0.4438 | No |
| 256 | HYLS1 |  |  | 1065 | 0.425 | 0.4438 | No |
| 257 | ARHGAP11B |  |  | 1069 | 0.424 | 0.4443 | No |
| 258 | CHEK2 |  |  | 1073 | 0.424 | 0.4447 | No |
| 259 | POC1A |  |  | 1080 | 0.421 | 0.4444 | No |
| 260 | JADE1 |  |  | 1089 | 0.419 | 0.4435 | No |
| 261 | NEK2 |  |  | 1100 | 0.417 | 0.4421 | No |
| 262 | ZNF714 |  |  | 1103 | 0.416 | 0.4428 | No |
| 263 | KCTD9 |  |  | 1104 | 0.416 | 0.4441 | No |
| 264 | CTDSPL2 |  |  | 1109 | 0.415 | 0.4443 | No |
| 265 | TDP1 |  |  | 1112 | 0.415 | 0.4450 | No |
| 266 | SRRT |  |  | 1121 | 0.414 | 0.4441 | No |
| 267 | RCC2 |  |  | 1124 | 0.413 | 0.4448 | No |
| 268 | CBX5 |  |  | 1132 | 0.412 | 0.4442 | No |
| 269 | UCP2 |  |  | 1138 | 0.410 | 0.4441 | No |
| 270 | BUB1 |  |  | 1145 | 0.408 | 0.4437 | No |
| 271 | TPRKB |  |  | 1168 | 0.403 | 0.4389 | No |
| 272 | POC5 |  |  | 1179 | 0.401 | 0.4374 | No |
| 273 | SCLT1 |  |  | 1201 | 0.397 | 0.4329 | No |
| 274 | GPD2 |  |  | 1202 | 0.397 | 0.4341 | No |
| 275 | MZT1 |  |  | 1219 | 0.393 | 0.4309 | No |
| 276 | PTMA |  |  | 1233 | 0.390 | 0.4286 | No |
| 277 | APOLD1 |  |  | 1239 | 0.389 | 0.4284 | No |
| 278 | PSMC3 |  |  | 1241 | 0.389 | 0.4293 | No |
| 279 | STMN1 |  |  | 1249 | 0.387 | 0.4286 | No |
| 280 | ESPL1 |  |  | 1255 | 0.386 | 0.4284 | No |
| 281 | ZNF93 |  |  | 1265 | 0.384 | 0.4271 | No |
| 282 | BUD13 |  |  | 1283 | 0.381 | 0.4237 | No |
| 283 | EFNB2 |  |  | 1306 | 0.377 | 0.4188 | No |
| 284 | NUP98 |  |  | 1314 | 0.375 | 0.4180 | No |
| 285 | STK17B |  |  | 1324 | 0.374 | 0.4167 | No |
| 286 | ANP32E |  |  | 1334 | 0.371 | 0.4154 | No |
| 287 | MAGOHB |  |  | 1336 | 0.369 | 0.4163 | No |
| 288 | RNASEH2A |  |  | 1341 | 0.369 | 0.4163 | No |
| 289 | DR1 |  |  | 1352 | 0.366 | 0.4147 | No |
| 290 | LDHA |  |  | 1374 | 0.361 | 0.4101 | No |
| 291 | ZNF85 |  |  | 1380 | 0.360 | 0.4098 | No |
| 292 | POLR2D |  |  | 1414 | 0.353 | 0.4018 | No |
| 293 | SPATA5 |  |  | 1424 | 0.352 | 0.4004 | No |
| 294 | TRAIP |  |  | 1430 | 0.351 | 0.4002 | No |
| 295 | KNSTRN |  |  | 1458 | 0.346 | 0.3938 | No |
| 296 | RBM15 |  |  | 1467 | 0.345 | 0.3927 | No |
| 297 | GTF3C5 |  |  | 1469 | 0.344 | 0.3935 | No |
| 298 | SRR |  |  | 1482 | 0.342 | 0.3912 | No |
| 299 | FUBP1 |  |  | 1519 | 0.337 | 0.3824 | No |
| 300 | CLIC1 |  |  | 1552 | 0.331 | 0.3746 | No |
| 301 | CDC27 |  |  | 1562 | 0.330 | 0.3732 | No |
| 302 | FUS |  |  | 1571 | 0.329 | 0.3720 | No |
| 303 | TMEM237 |  |  | 1576 | 0.328 | 0.3719 | No |
| 304 | CALM2 |  |  | 1625 | 0.320 | 0.3597 | No |
| 305 | IVNS1ABP |  |  | 1628 | 0.320 | 0.3602 | No |
| 306 | KARS |  |  | 1632 | 0.319 | 0.3603 | No |
| 307 | ARHGAP19 |  |  | 1658 | 0.316 | 0.3545 | No |
| 308 | G3BP1 |  |  | 1661 | 0.315 | 0.3549 | No |
| 309 | LSM4 |  |  | 1676 | 0.313 | 0.3520 | No |
| 310 | TMX1 |  |  | 1703 | 0.310 | 0.3458 | No |
| 311 | HMGB1 |  |  | 1720 | 0.308 | 0.3424 | No |
| 312 | ZMYM1 |  |  | 1737 | 0.306 | 0.3389 | No |
| 313 | NUP93 |  |  | 1778 | 0.301 | 0.3289 | No |
| 314 | RUVBL2 |  |  | 1779 | 0.301 | 0.3298 | No |
| 315 | ENOSF1 |  |  | 1823 | 0.296 | 0.3189 | No |
| 316 | CNTRL |  |  | 1830 | 0.294 | 0.3182 | No |
| 317 | ILF2 |  |  | 1833 | 0.294 | 0.3185 | No |
| 318 | SRSF3 |  |  | 1834 | 0.294 | 0.3195 | No |
| 319 | RAB8A |  |  | 1857 | 0.291 | 0.3143 | No |
| 320 | UBAP2 |  |  | 1877 | 0.289 | 0.3100 | No |
| 321 | ODF2 |  |  | 1887 | 0.287 | 0.3084 | No |
| 322 | QSER1 |  |  | 1922 | 0.282 | 0.2999 | No |
| 323 | NUP37 |  |  | 1929 | 0.281 | 0.2992 | No |
| 324 | E2F3 |  |  | 1961 | 0.277 | 0.2915 | No |
| 325 | PIP4K2A |  |  | 1981 | 0.275 | 0.2871 | No |
| 326 | DHX15 |  |  | 1985 | 0.275 | 0.2872 | No |
| 327 | TRIM26 |  |  | 2005 | 0.272 | 0.2828 | No |
| 328 | FAM122B |  |  | 2009 | 0.271 | 0.2828 | No |
| 329 | CENPT |  |  | 2037 | 0.268 | 0.2762 | No |
| 330 | SKA2 |  |  | 2061 | 0.266 | 0.2707 | No |
| 331 | MDM1 |  |  | 2080 | 0.264 | 0.2666 | No |
| 332 | MPHOSPH9 |  |  | 2082 | 0.264 | 0.2672 | No |
| 333 | EDRF1 |  |  | 2108 | 0.262 | 0.2611 | No |
| 334 | CEP192 |  |  | 2109 | 0.262 | 0.2619 | No |
| 335 | LDLR |  |  | 2128 | 0.260 | 0.2578 | No |
| 336 | CKAP5 |  |  | 2135 | 0.258 | 0.2569 | No |
| 337 | ARHGEF39 |  |  | 2168 | 0.255 | 0.2489 | No |
| 338 | XRCC6 |  |  | 2180 | 0.254 | 0.2467 | No |
| 339 | C2orf69 |  |  | 2183 | 0.254 | 0.2469 | No |
| 340 | ANXA5 |  |  | 2187 | 0.253 | 0.2469 | No |
| 341 | SEPHS1 |  |  | 2188 | 0.253 | 0.2477 | No |
| 342 | PKM |  |  | 2199 | 0.252 | 0.2457 | No |
| 343 | CBX1 |  |  | 2210 | 0.251 | 0.2437 | No |
| 344 | GPC6 |  |  | 2232 | -0.251 | 0.2387 | No |
| 345 | PTPRE |  |  | 2265 | -0.255 | 0.2307 | No |
| 346 | OSBPL8 |  |  | 2269 | -0.256 | 0.2307 | No |
| 347 | ZNF24 |  |  | 2310 | -0.260 | 0.2205 | No |
| 348 | NEUROD1 |  |  | 2312 | -0.261 | 0.2211 | No |
| 349 | EFS |  |  | 2326 | -0.262 | 0.2183 | No |
| 350 | MSI1 |  |  | 2379 | -0.268 | 0.2048 | No |
| 351 | DCAF16 |  |  | 2396 | -0.270 | 0.2013 | No |
| 352 | CDON |  |  | 2454 | -0.278 | 0.1865 | No |
| 353 | TMEM158 |  |  | 2476 | -0.280 | 0.1816 | No |
| 354 | MID1 |  |  | 2486 | -0.283 | 0.1800 | No |
| 355 | RAB11A |  |  | 2491 | -0.283 | 0.1798 | No |
| 356 | CDK19 |  |  | 2543 | -0.292 | 0.1667 | No |
| 357 | HMGN3 |  |  | 2561 | -0.295 | 0.1629 | No |
| 358 | SRGAP2C |  |  | 2628 | -0.305 | 0.1457 | No |
| 359 | ARID2 |  |  | 2644 | -0.307 | 0.1425 | No |
| 360 | KIAA1191 |  |  | 2645 | -0.307 | 0.1435 | No |
| 361 | CBFA2T2 |  |  | 2646 | -0.308 | 0.1445 | No |
| 362 | HSP90B1 |  |  | 2648 | -0.308 | 0.1451 | No |
| 363 | MIB1 |  |  | 2662 | -0.310 | 0.1425 | No |
| 364 | SMARCD1 |  |  | 2665 | -0.311 | 0.1429 | No |
| 365 | GNG4 |  |  | 2702 | -0.316 | 0.1340 | No |
| 366 | SOGA1 |  |  | 2731 | -0.322 | 0.1273 | No |
| 367 | CCDC167 |  |  | 2753 | -0.327 | 0.1226 | No |
| 368 | GAS2L3 |  |  | 2755 | -0.327 | 0.1233 | No |
| 369 | HSPA13 |  |  | 2773 | -0.331 | 0.1197 | No |
| 370 | CPEB2 |  |  | 2787 | -0.333 | 0.1171 | No |
| 371 | ATCAY |  |  | 2865 | -0.346 | 0.0971 | No |
| 372 | ZNF43 |  |  | 2872 | -0.348 | 0.0965 | No |
| 373 | FIGN |  |  | 2879 | -0.351 | 0.0959 | No |
| 374 | ZNF292 |  |  | 2882 | -0.352 | 0.0965 | No |
| 375 | RSRC1 |  |  | 2966 | -0.367 | 0.0748 | No |
| 376 | SERF2 |  |  | 2975 | -0.368 | 0.0737 | No |
| 377 | TIMM8B |  |  | 2980 | -0.369 | 0.0738 | No |
| 378 | PPP4R2 |  |  | 2989 | -0.370 | 0.0727 | No |
| 379 | NUDCD2 |  |  | 3003 | -0.373 | 0.0703 | No |
| 380 | FBXL5 |  |  | 3034 | -0.381 | 0.0633 | No |
| 381 | PAX6 |  |  | 3055 | -0.387 | 0.0590 | No |
| 382 | YAF2 |  |  | 3061 | -0.388 | 0.0588 | No |
| 383 | TMSB4X |  |  | 3141 | -0.412 | 0.0384 | No |
| 384 | PIK3R3 |  |  | 3151 | -0.414 | 0.0372 | No |
| 385 | GOLIM4 |  |  | 3154 | -0.414 | 0.0379 | No |
| 386 | DZIP3 |  |  | 3159 | -0.416 | 0.0381 | No |
| 387 | HMGN5 |  |  | 3173 | -0.421 | 0.0358 | No |
| 388 | MIDN |  |  | 3175 | -0.421 | 0.0369 | No |
| 389 | NFIC |  |  | 3194 | -0.427 | 0.0333 | No |
| 390 | HEG1 |  |  | 3200 | -0.429 | 0.0332 | No |
| 391 | ANP32A |  |  | 3217 | -0.432 | 0.0302 | No |
| 392 | CHRDL1 |  |  | 3219 | -0.432 | 0.0312 | No |
| 393 | NARF |  |  | 3223 | -0.434 | 0.0317 | No |
| 394 | LAPTM4A |  |  | 3231 | -0.436 | 0.0312 | No |
| 395 | ZBTB18 |  |  | 3239 | -0.438 | 0.0306 | No |
| 396 | NDUFAF3 |  |  | 3286 | -0.452 | 0.0194 | No |
| 397 | ELAVL3 |  |  | 3341 | -0.472 | 0.0060 | No |
| 398 | MARCKS |  |  | 3369 | -0.487 | 0.0001 | No |
| 399 | TULP4 |  |  | 3398 | -0.497 | -0.0061 | No |
| 400 | SYF2 |  |  | 3419 | -0.509 | -0.0100 | No |
| 401 | NUDT4 |  |  | 3421 | -0.510 | -0.0087 | No |
| 402 | DANCR |  |  | 3428 | -0.511 | -0.0087 | No |
| 403 | COX17 |  |  | 3461 | -0.524 | -0.0159 | No |
| 404 | H3F3A |  |  | 3471 | -0.528 | -0.0167 | No |
| 405 | JAM2 |  |  | 3477 | -0.532 | -0.0165 | No |
| 406 | CLVS2 |  |  | 3547 | -0.569 | -0.0336 | No |
| 407 | ARL6IP1 |  |  | 3574 | -0.586 | -0.0390 | No |
| 408 | ZEB1 |  |  | 3589 | -0.598 | -0.0410 | No |
| 409 | AGO1 |  |  | 3611 | -0.609 | -0.0448 | No |
| 410 | DACH1 |  |  | 3614 | -0.613 | -0.0435 | No |
| 411 | PHLDA1 |  |  | 3622 | -0.617 | -0.0435 | No |
| 412 | BLOC1S1 |  |  | 3630 | -0.627 | -0.0435 | No |
| 413 | TFDP2 |  |  | 3637 | -0.634 | -0.0432 | No |
| 414 | CCNG2 |  |  | 3650 | -0.648 | -0.0444 | No |
| 415 | SMIM14 |  |  | 3654 | -0.651 | -0.0432 | No |
| 416 | TMEM256 |  |  | 3673 | -0.662 | -0.0461 | No |
| 417 | TRIM2 |  |  | 3688 | -0.672 | -0.0479 | No |
| 418 | MPPED2 |  |  | 3701 | -0.685 | -0.0491 | No |
| 419 | STON2 |  |  | 3702 | -0.689 | -0.0469 | No |
| 420 | MDK |  |  | 3706 | -0.691 | -0.0456 | No |
| 421 | LCORL |  |  | 3718 | -0.698 | -0.0465 | No |
| 422 | TGIF1 |  |  | 3728 | -0.707 | -0.0467 | No |
| 423 | GTF2I |  |  | 3749 | -0.727 | -0.0500 | No |
| 424 | PLCB1 |  |  | 3779 | -0.769 | -0.0556 | No |
| 425 | ENC1 |  |  | 3787 | -0.774 | -0.0551 | No |
| 426 | PTMS |  |  | 3791 | -0.780 | -0.0535 | No |
| 427 | ZNF608 |  |  | 3810 | -0.798 | -0.0560 | No |
| 428 | CDKN1B |  |  | 3812 | -0.800 | -0.0538 | No |
| 429 | MYEF2 |  |  | 3818 | -0.807 | -0.0526 | No |
| 430 | FJX1 |  |  | 3824 | -0.813 | -0.0515 | No |
| 431 | MAGI1 |  |  | 3827 | -0.815 | -0.0495 | No |
| 432 | NKAIN3 |  |  | 3851 | -0.845 | -0.0532 | No |
| 433 | DAAM1 |  |  | 3856 | -0.849 | -0.0517 | No |
| 434 | LDLRAD3 |  |  | 3857 | -0.849 | -0.0490 | No |
| 435 | MEX3A |  |  | 3866 | -0.867 | -0.0485 | No |
| 436 | ROBO2 |  |  | 3879 | -0.899 | -0.0490 | No |
| 437 | CHD7 |  |  | 3880 | -0.904 | -0.0462 | No |
| 438 | SORT1 |  |  | 3891 | -0.929 | -0.0461 | No |
| 439 | DDAH2 |  |  | 3895 | -0.936 | -0.0440 | No |
| 440 | FBLN1 |  |  | 3909 | -0.965 | -0.0446 | No |
| 441 | LRRN1 |  |  | 3913 | -0.970 | -0.0424 | No |
| 442 | STK17A |  |  | 3925 | -1.012 | -0.0423 | No |
| 443 | TMTC4 |  |  | 3933 | -1.023 | -0.0410 | No |
| 444 | CUX1 |  |  | 3955 | -1.079 | -0.0434 | No |
| 445 | HES6 |  |  | 3961 | -1.092 | -0.0414 | No |
| 446 | FRMD4B |  |  | 3972 | -1.116 | -0.0407 | No |
| 447 | MYO10 |  |  | 3990 | -1.193 | -0.0417 | No |
| 448 | CSRP2 |  |  | 4000 | -1.238 | -0.0403 | No |
| 449 | SESN3 |  |  | 4003 | -1.260 | -0.0370 | No |
| 450 | NFIX |  |  | 4004 | -1.262 | -0.0330 | No |
| 451 | NFIB |  |  | 4007 | -1.310 | -0.0295 | No |
| 452 | NOVA1 |  |  | 4008 | -1.312 | -0.0254 | No |
| 453 | SLCO5A1 |  |  | 4015 | -1.362 | -0.0229 | No |
| 454 | INSM1 |  |  | 4059 | -1.733 | -0.0293 | No |
| 455 | ASCL1 |  |  | 4061 | -1.762 | -0.0241 | No |
| 456 | LINC00461 |  |  | 4068 | -1.907 | -0.0198 | No |
| 457 | POU3F2 |  |  | 4071 | -2.024 | -0.0141 | No |
| 458 | CKB |  |  | 4075 | -2.149 | -0.0083 | No |
| 459 | NFIA |  |  | 4078 | -2.210 | -0.0019 | No |
| 460 | ID4 |  |  | 4079 | -2.308 | 0.0052 | No |
Table: GSEA details [plain text format]

  

Fig 2: RICHARDS\_NOWAKOWSKI\_SCIENCE\_2017\_IPC\_DIV1\_UPREG: Random ES distribution      
 Gene set null distribution of ES for **RICHARDS\_NOWAKOWSKI\_SCIENCE\_2017\_IPC\_DIV1\_UPREG**
